# Neurobiology and neuroimaging of the maternal brain

**DOI:** 10.1192/j.eurpsy.2023.1351

**Published:** 2023-07-19

**Authors:** A.-L. Sutter-Dallay

**Affiliations:** Perinatal psychiatry, CH Charles Perrens and Bordeaux University, Bordeaux, France

## Abstract

**Introduction:**

The epidemiology of psychiatric disorders of the perinatal period and their mainly thymic dimension are now well established. However, the underlying physiopathology remains uncertain and poorly explored.

**Objectives:**

The purpose of this presentation is to explore the current knowledge in terms of neurobiology/neuroimaging underlying the modifications in maternal brain functioning and the links with perinatal psychiatric disorders.

**Methods:**

A narrative review of the current international literature was carried out.

**Results:**

Results of the current studies suggest that during pregnancy and the postpartum period, hormones and sensory interactions with the offspring relate to complex structural and functional changes in the brain. This reproduction-related brain plasticity embraces various areas implicated in maternal caregiving, primarily regions involved in reward/motivation, salience/threat detection, emotional regulation, and social cognition such as the ability to empathize and infer the mental state of the baby. Some structural irregularities and differences in activation patterns potentially involved in the triggering of disorders are starting to be identified.

**Conclusions:**

The survival of newborns is largely dependent on the mother, and her brain appears to have evolved to support mother-infant bonding and sensitive care. Brain research offers a growing scientific understanding of the neural correlates of these disorders and opens a window to their prevention and treatment.

**Disclosure of Interest:**

None Declared

